# Supplementary material for: Microbial Detection and Quantification of Low-Biomass Water Samples Using an International Space Station Smart Sample Concentrator
Source: Microorganisms. 2023 Sep 13;11(9):2310. doi: 10.3390/microorganisms11092310 (PMC10537578; doi:10.3390/microorganisms11092310)
Supplement: Supplementary file 1 [file microorganisms-11-02310-s001.zip › microorganisms-2534595-supplementary-figures.pdf]

1. Supplementary Figures

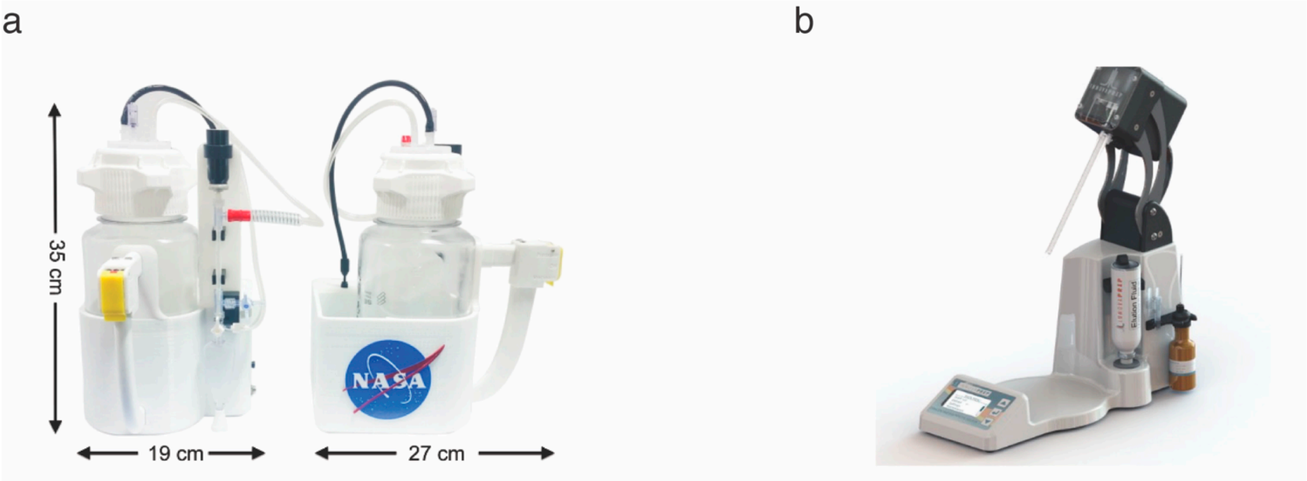

Figure S1. The International Space Station Smart Sample Concentrator (iSSC) (a) and Concentrating Pipette Select (b). Presented are concentrating devices developed by InnovaPrep for application on Earth (b) and in microgravity (a).

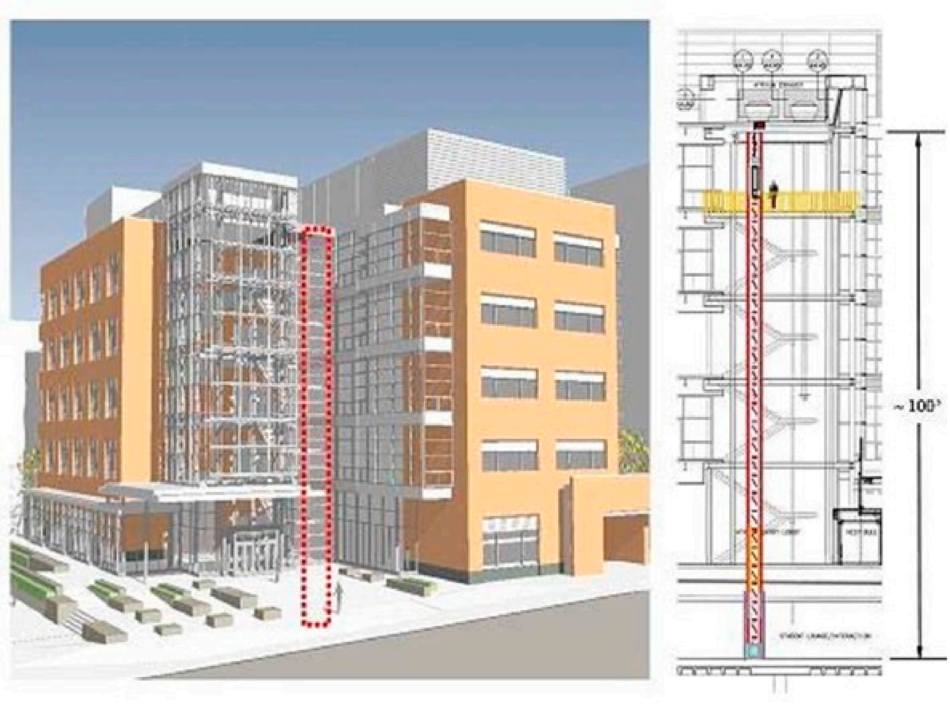

Figure S2. The Dryden drop tower set up. Presented is the setup of the Dryden drop tower used for testing the iSSC microgravity compatibility.

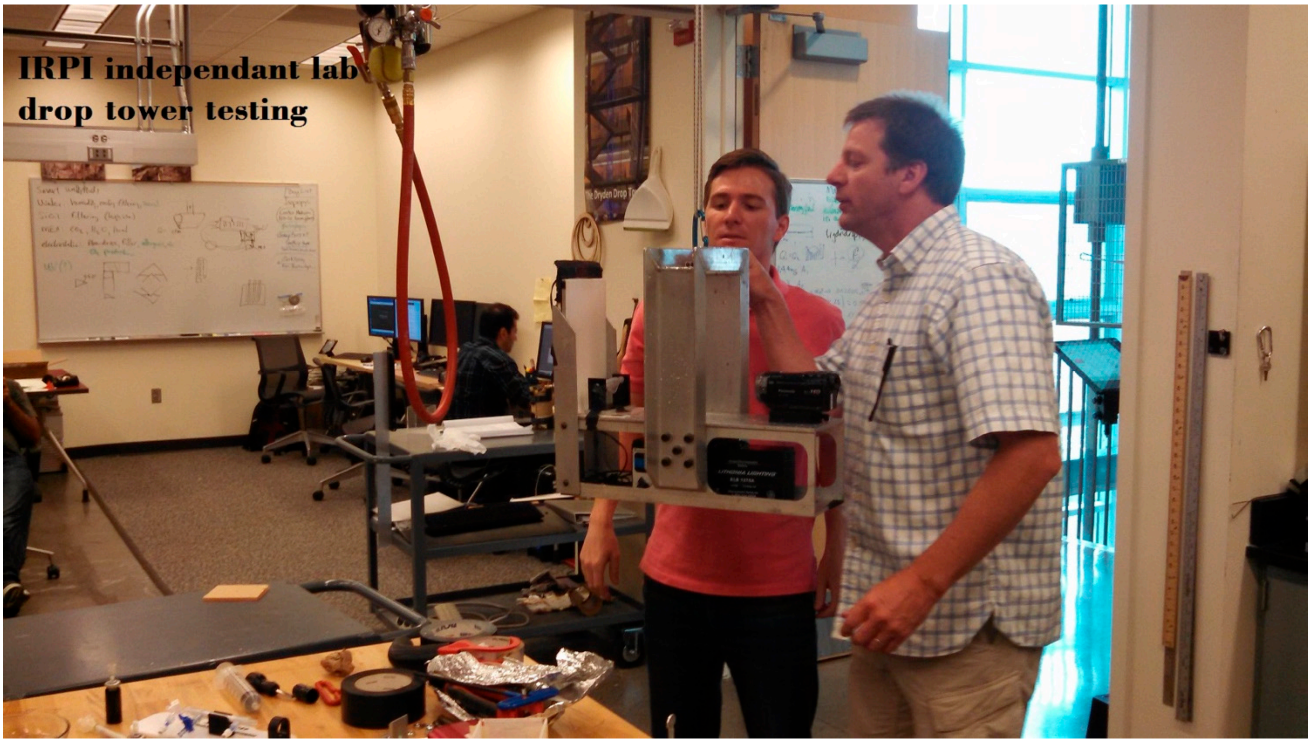

Figure S3. IRPI independent lab drop tower testing.

2. Supplementary Tables

Supplementary Table 1. qPCR results for *Sphingomonas paucimobilis, Ralstonia pickettii* and *Cupriavidus basilensis*
Supplementary Table 2. CFU results for *Sphingomonas paucimobilis, Ralstonia pickettii* and *Cupriavidus basilensis*
Excel files provided

3. Supplementary Videos

Supplementary Video 1. IRPI Dryden Drop Tower. The video shows the drop of the iSSC system during the impact testing.
Supplementary Video 2. Volume comparison. The vieo shows the comparison of the volumes and their behavior during the drop.
Supplementary Video 3. The behavior of liquid in a single CFAC during the impact. The video shows the behavior of the liquid during the drop in a single CFAC.
Supplementary Video 4. The behavior of liquid in a single CFAC during the impact in different orientations. The video shows the behavior of the liquid during the drop in a single CFAC in different orientations.
Video files provided
