# Supplementary material for: Microbial Detection and Quantification of Low-Biomass Water Samples Using an International Space Station Smart Sample Concentrator
Source: Microorganisms. 2023 Sep 13;11(9):2310. doi: 10.3390/microorganisms11092310 (PMC10537578; doi:10.3390/microorganisms11092310)
Supplement: Supplementary file 1 [file microorganisms-11-02310-s001.zip › Supplementary File 1 WL2-Parra Final.pdf]

National Aeronautics and Space Administration (NASA)
Ames Research Center
Moffett Field, CA 94035-1000

SCIENCE/FLUIDICS REPORT

**Title**: WetLab-2 Parra Final Report	**Report #:** WL2-SCI-20181019
**Project**: WetLab-2	**Category/Subcategory:** Science & Fluidics
**Prepared by:** Macarena Parra	**Issue Date: 10/19/18**

Purpose

To document the results of the WetLab-2 Parra investigation: To test the functionality of a passive method to remove air bubbles from a liquid sample (e.g., extracted RNA) and draw into a repeater pipette. This would simplify the workflow of the Wet Lab-2 facility currently on the ISS, as well as reduce up-mass and crew time.

Background

We have been working to simplify the workflow and reduce the needed crew time of the WetLab-2 system. One potential simplification is to replace the current Pipette Loader, used to remove air bubbles from extracted RNA and load the pipette tip, with a passive module requiring less crew manipulation and stowage mass/volume. The proposed Debubbler leverages off the same surface tension-driven fluidic design principles used in the zero gravity coffee cup, and more recently demonstrated in parabolic flight testing of a similar module in development by InnovaPrep for use in conjunction with the Microbial Monitoring concentrator and potentially WL. This device will accept a liquid/gas mixture, then wick bubble-free fluid to a pipette tip interface for drawing in sample or reagents. See Figure 1.

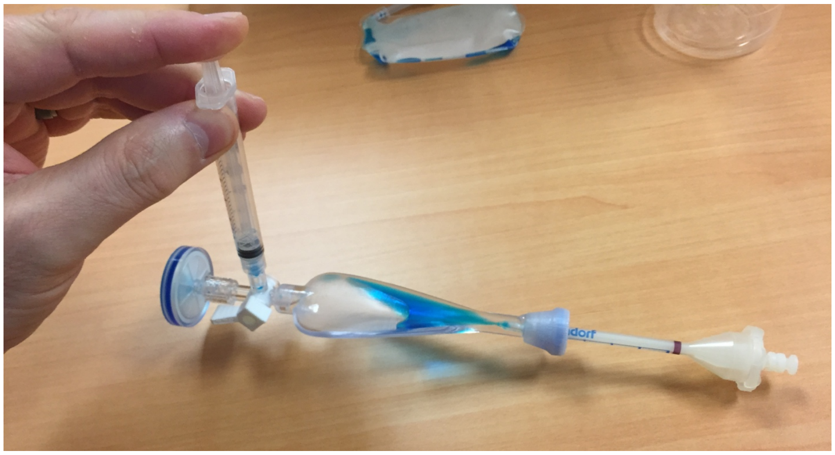

Fig 1. Debubbler

Plan

The WetLab-2 Parra investigation consisted of two runs:

1. Basic Run: Demonstrate the basic ability to debubble a model liquid/gas mixture and draw into a repeater pipette for dispensing into empty SmartTubes. A mix of dye-colored buffer and air was employed. After drawing debubbled fluid into the pipette, the tip was

visually examined. Fluid was then dispensed to eight SmartTubes and spun on a WL2 rotor using an ISS cordless drill to position the fluid in the tube detection windows. Tubes were photographed to quantify the dispensed volume.

2. Enhanced Run: Demonstrate the same debubbling pipette loading performance with a DNA solution and perform qPCR analysis using the SmartCycler. As in the Basic Run, a DNA solution/air mixture was debubbled and dispensed into SmartTubes, this time containing lyophilized reagents for qPCR analysis. The quality of the data was judged by the smoothness of the qPCR curves (bubble formation during thermocycling introduces "choppiness" into the curves) and the uniformity between replicate tubes.

Test Procedures

Crew Procedures 2.001 WET LAB-2 PARRA BASIC SESSION and 2.002 WET LAB-2 PARRA ENHANCED SESSION were followed for in-flight operations. For the Enhanced Run, a ground control was also performed later that day. The ground control consisted of running steps 6-14 of the procedure except that no photos of the SmartTubes were taken.

Notes from Basic Session:

- During Run 1, Ricky was unable to pull a vacuum on the Debubbler, he tried twice. Since this step was an optional safeguard that was not required, we dropped it from later runs with no issue.
- During Run 1, it looked like the tubes were not properly spun (the spin occurred during LOS) so we asked him to repeat the 30 sec spin. There were no problems during later spins.
- During Run 2, some liquid went onto the filter when the valve was turned so a wrist-flick was added to future runs to minimize the chance and amount of fluids wetting the filter.
- During a number of the runs there were some bubbles seen at Debubbler/Combitip interface. These were transferred to the tip of Combitip after filling, but were easily removed with a dispense or two from the Combitip before tube loading (as per procedure).

Notes from Enhanced Session:

- One deviation from the procedure was that Ricky had not retrieved the cold stowage items at the start of the procedure, instead he retrieved them just before starting the Debubbler ops.
- For the ground control, items were removed from 4C just before starting Debubbler ops to match the on-orbit ops just in case the colder reagents had an effect on the Debubbler ops or the SmartCycler run.
- The vacuum priming step during Debubbler use was skipped, as done in second and third repeats in the Basic session

Volume Test:

A ground test was conducted to quantify the effect of small differences in loading volume on quantitative PCR results. SmartTubes are nominally filled with 25ul. Spare SmartTubes

containing lyophilized reagents were filled with varying volumes of the DNA solution from 23ul to 30ul in 1ul increments and qPCR reactions were run.

Results:

<u>Basic Run</u>

Visual observations of the Combitip during the Basic Run identified only small bubbles located near the tip; these were easily removed by performing a few dispenses into the Debubbler before filling tubes. Photos of the SmartTubes from the Basic Runs showed no bubbles in the tubes and only minor differences in loading volumes (Figure 2). Figure 3 shows Tubes filled with 20, 25, and 30 ul and serves as a good reference for the volumes loaded in each tube in flight. The different volumes in the tubes is likely due to some variation in the extent of manual dispense lever actuation on the Repeater Pipette.

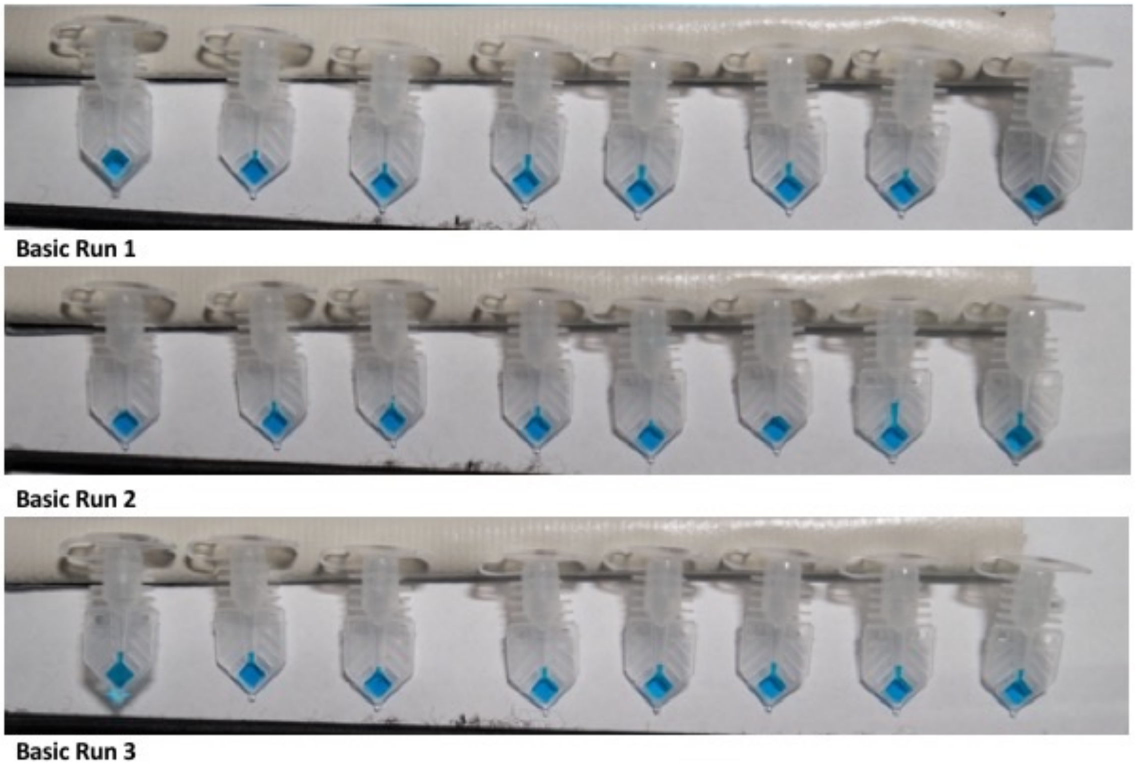

Figure 2. Photos of tubes from Basic Runs

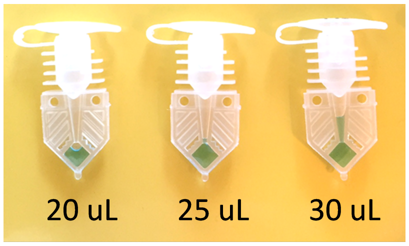

Fig 3. Tubes filled with different volumes of liquid

Volume Test

Results from the volume test conducted on the ground are shown in Table 1. There was no difference in Ct values when different volumes were added to the tubes confirming that small differences in loading volume should not affect the results.

Volume	Ct (Avg)	SD
23	21.78	0.0990
24	21.785	0.0071
25	22.26	0.2404
26	21.825	0.3182
27	22.14	0.0424
28	21.925	0.0495
29	21.905	0.1202
30	21.915	0.2333
**Total**	**21.942**	**0.209**

Table 1. Results from volume test.

Enhanced Run

Photos of the SmartTubes were taken before and after the qPCR run and are shown in Figure 4. Results of the on-orbit qPCR run are shown in Figure 5 and those from the ground control are in Figure 6. The PCR curve from flight Tube 11 shows some choppiness, likely due to very fine bubbles. Maximum fluorescence in the flight tubes is lower than in the ground tubes, this matches data from previous flight experiments.

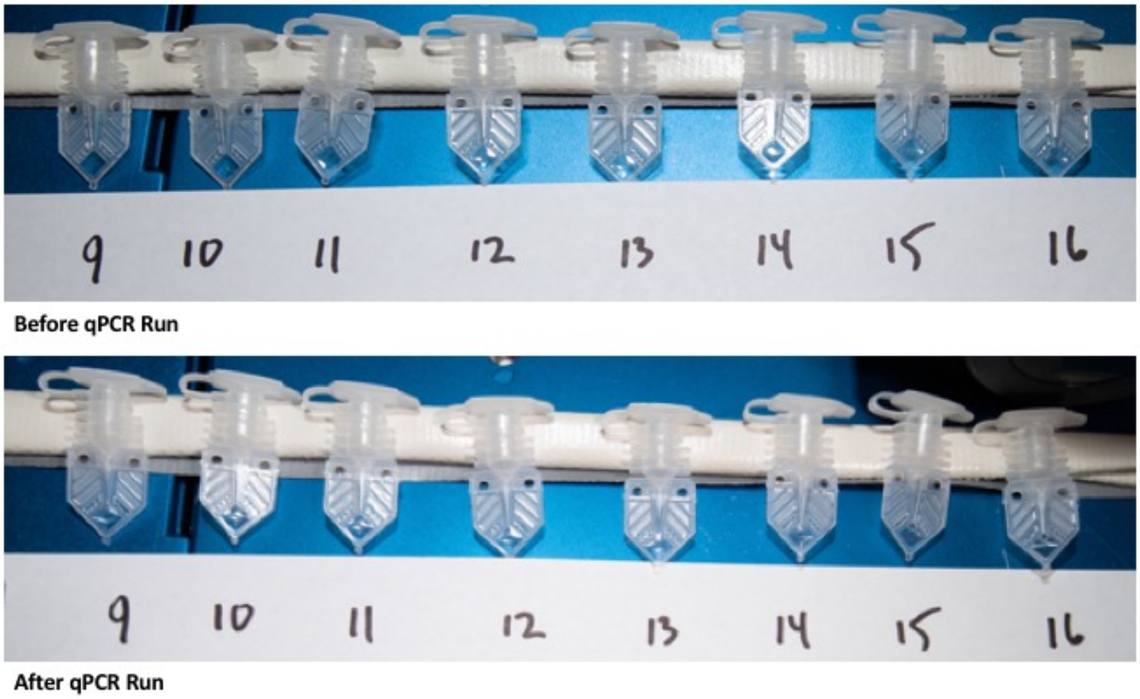

Figure 4. Photos of tubes Before and After the qPCR run from the Enhanced Run

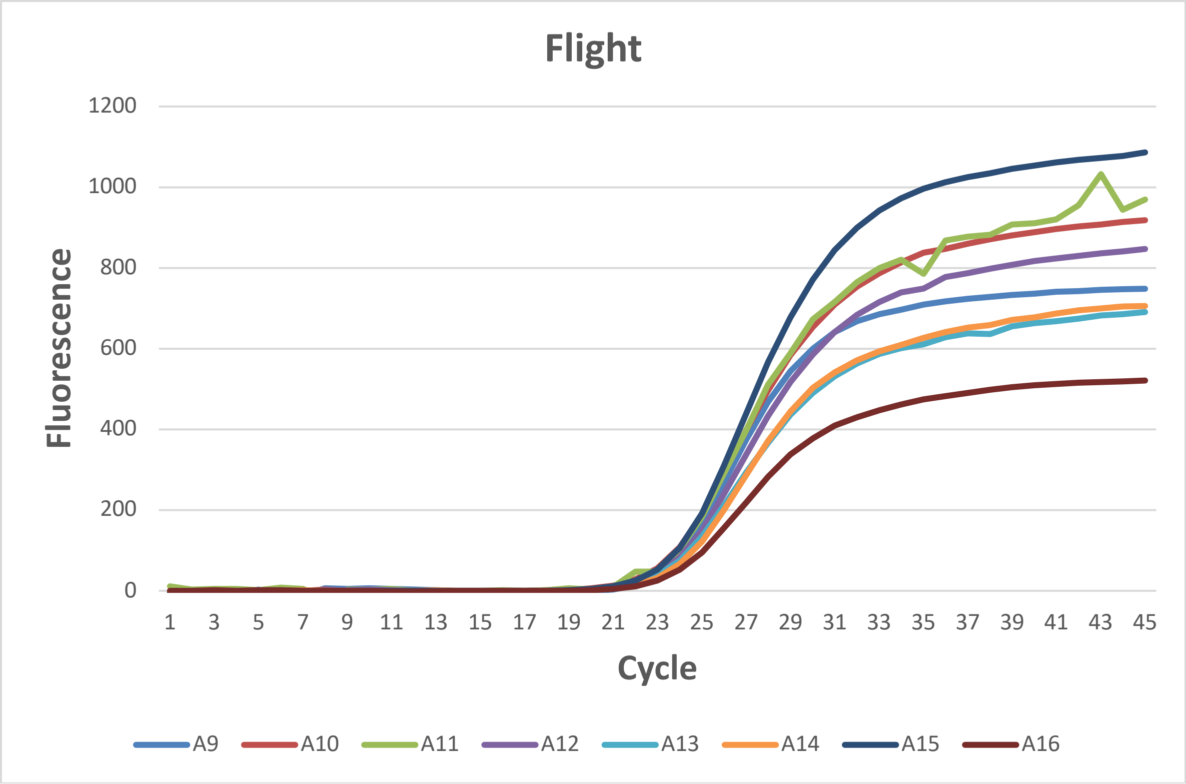

Figure 5. Flight amplification curves.

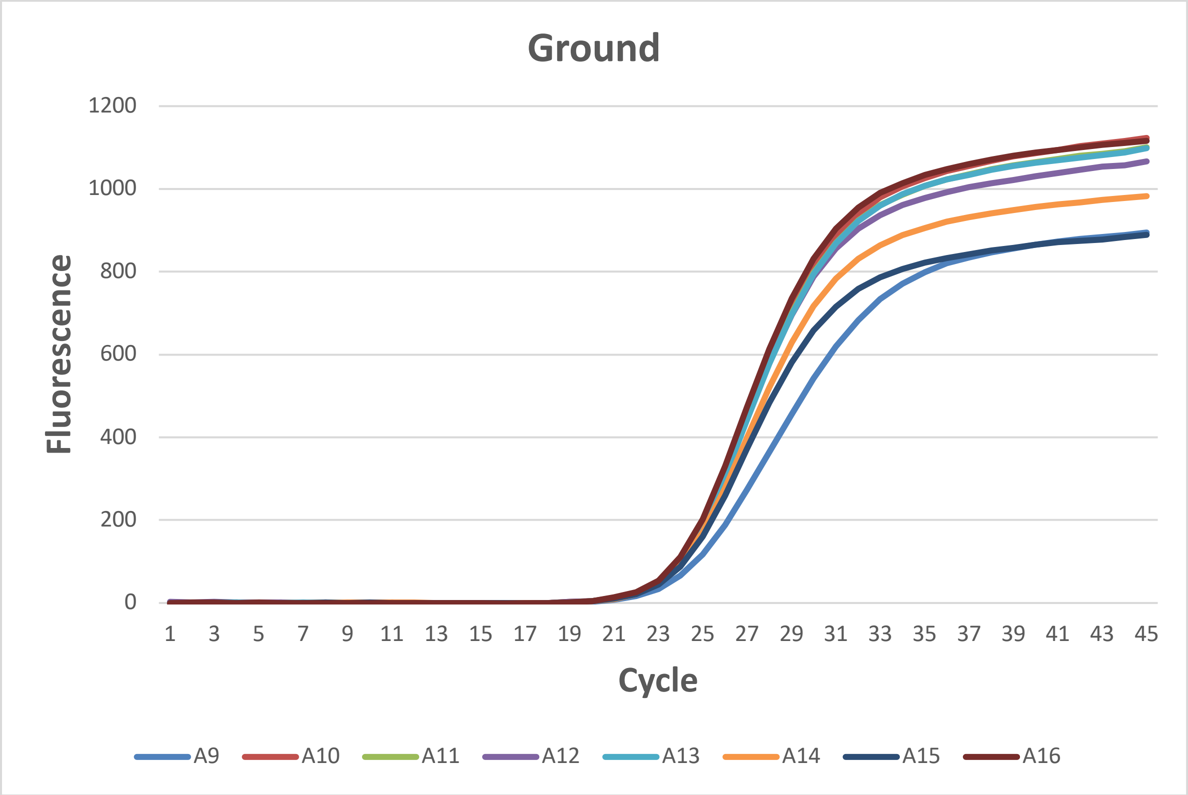

Figure 6. Ground control amplification curves.

Tube 11 in the flight data in Figure 6 shows some "choppiness" typically associated with bubbles migrating in the excitation/detection light path within the tube. However, this choppiness does not correlate with the few bubbles seen in photographs of the tubes before the PCR run (for example near the center of the tube 15 diamond-shaped detection widow in Figure 7), which are likely a remnant of the lyophilized assay wet-out. Note that tube 11 has no signs of such bubbles before the PCR run. The choppiness in the PCR curve of tube 11 is instead most likely due to the very fine bubbles seen clinging to the bottom right edge of the detection window (Figure 7). Since these bubbles were not present before the qPCR run was started, they were likely formed by degassing during the repeated heating/cooling cycles of the PCR amplification.

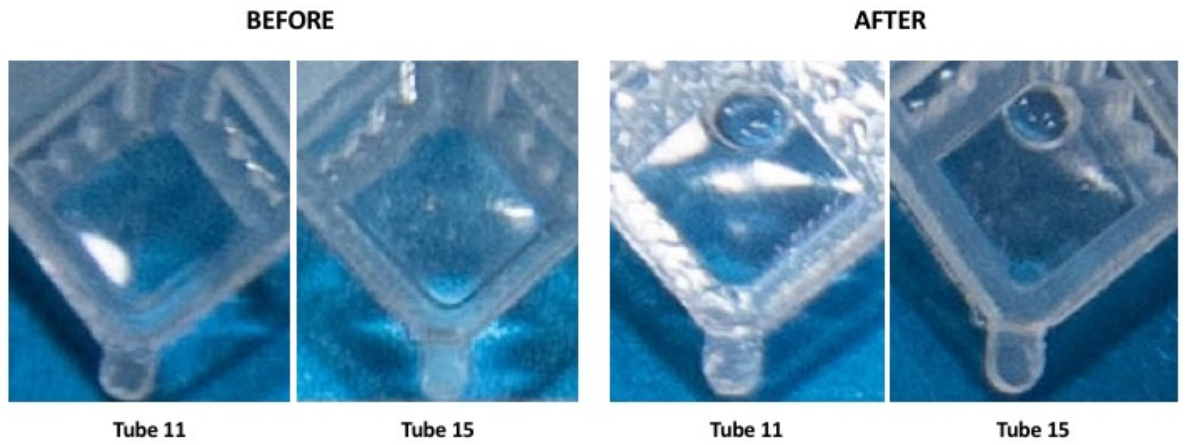

Figure 7. Close-ups of tubes 11 and 15

Ct values from flight and ground runs are listed in Table 2. While there is more variability in the flight Cts, they match results from the ground runs very closely.

Tube #	Flight Ct	Ground Ct
9	22.63	22.77
10	22.04	22.2
11	21.53	22.18
12	22.58	22.2
13	22.58	22.28
14	22.88	22.33
15	22.16	22.38
**Average**	**22.45**	**22.31**
**SD**	**0.52**	**0.20**

Table 2. Ct values from Flight and Ground.

Conclusions

Results from the flight operations show that the Debubbler can be used instead of the Pipette Loader for future WetLab-2 operations. This will result in lower crew time needs, lower up-mass and lower costs.

Signature: Macarena Parra Date: 10/19/18
